# Test-retest reliability and construct validity of the ENERGY-child questionnaire on energy balance-related behaviours and their potential determinants: the ENERGY-project

**DOI:** 10.1186/1479-5868-8-136

**Published:** 2011-12-09

**Authors:** Amika S Singh, Froydis N Vik, Mai JM Chinapaw, Léonie Uijtdewilligen, Maïté Verloigne, Juan M Fernández-Alvira, Sarolta Stomfai, Yannis Manios, Marloes Martens, Johannes Brug

**Affiliations:** 1 VU University Medical Center, EMGO Institute for Health and Care Research, Dpt. of Public and Occupational Health, Amsterdam, the Netherlands; 2 University of Agder, Department of Public Health, Sport and Nutrition, Kristiansand, Norway; 3 Ghent University, Department of Movement and Sport Sciences, Gent, Belgium; 4 GENUD (Growth, Exercise, NUtrition and Development) Research Group, Escuela Universitaria de Ciencias de la Salud, Universidad de Zaragoza, Zaragoza, Spain; 5 University of Pécs, Department of Paediatrics, Pecs, Hungary; 6 Harokopio University, Department of Nutrition and Dietetics, Athens, Greece; 7 ResCon, Amsterdam, the Netherlands; 8 VU University Medical Center, EMGO Institute for Health and Care Research, Amsterdam, the Netherlands

**Keywords:** child questionnaire, self-report, psychometric, physical activity, sedentary behaviour, soft drinks, fruit juice, active transport, breakfast

## Abstract

**Background:**

Insight in children's energy balance-related behaviours (EBRBs) and their determinants is important to inform obesity prevention research. Therefore, reliable and valid tools to measure these variables in large-scale population research are needed.

**Objective:**

To examine the test-retest reliability and construct validity of the child questionnaire used in the ENERGY-project, measuring EBRBs and their potential determinants among 10-12 year old children.

**Methods:**

We collected data among 10-12 year old children (n = 730 in the test-retest reliability study; n = 96 in the construct validity study) in six European countries, i.e. Belgium, Greece, Hungary, the Netherlands, Norway, and Spain. Test-retest reliability was assessed using the intra-class correlation coefficient (ICC) and percentage agreement comparing scores from two measurements, administered one week apart. To assess construct validity, the agreement between questionnaire responses and a subsequent face-to-face interview was assessed using ICC and percentage agreement.

**Results:**

Of the 150 questionnaire items, 115 (77%) showed good to excellent test-retest reliability as indicated by ICCs > .60 or percentage agreement ≥ 75%. Test-retest reliability was moderate for 34 items (23%) and poor for one item. Construct validity appeared to be good to excellent for 70 (47%) of the 150 items, as indicated by ICCs > .60 or percentage agreement ≥ 75%. From the other 80 items, construct validity was moderate for 39 (26%) and poor for 41 items (27%).

**Conclusions:**

Our results demonstrate that the ENERGY-child questionnaire, assessing EBRBs of the child as well as personal, family, and school-environmental determinants related to these EBRBs, has good test-retest reliability and moderate to good construct validity for the large majority of items.

## Background

Energy balance-related behaviours (EBRBs), i.e. lack of physical activity, excess sedentary behaviour and unhealthy dietary patterns are considered to be important contributors to the obesity epidemic [1]. In order to adequately inform prevention and intervention research on lifestyle behaviours, the assessment of EBRBs and their personal and environmental correlates and potential determinants is of utmost importance.

Large-scale observational and intervention studies most often have to rely on questionnaires to assess lifestyle behaviours and their potential determinants: questionnaire assessments are inexpensive, easy to administer and are widely accepted by study participants [2,3]. However, questionnaire assessments rely on self-report that may be prone to recall and social desirability bias [4].

In a recent review Lubans *et al*. [5] concluded that self-report measures can provide reliable estimates of screen time in children and adolescents. However, the validity of these questionnaires remains largely untested. A review of physical activity questionnaires in young people by Chinapaw *et al*. [6] concluded that there was no physical activity questionnaire with both acceptable validity as well as reliability. Thus, more high-quality research is required into the measurement properties of measurement instruments of sedentary behaviour and physical activity in young people [6].

No gold standard exists for the assessment of dietary intake in large research populations. The commonly used methods in larger populations include food records, food frequency questionnaires, or 24-hour recalls all relying on self-report. All of these suffer from bias due to over- or underreporting and little is known to what extent factors like for example age, cognition, social background and complexity of questions influence the outcomes of the dietary assessment in children [7,8].

Even less research has been conducted on the psychometric characteristics of measures of determinants of EBRBs [9]. Moreover, most questionnaires regarding energy-balance behaviours and potential behavioural determinants have been developed for administration in specific countries, while, especially in Europe, cross country studies and comparisons are now common and supported by the European Commission's framework programs.

It can be concluded that reliable and valid questionnaires in the area of potential drivers of childhood overweight and obesity are scarce, especially those covering a range of energy balance-related behaviours that can be used in large-scale studies across countries.

The ENERGY-project is a European Commission funded cross-European project to gain more insight in EBRBs and their potential behavioural determinants, and to inform and test a school-based and family-involved obesity prevention intervention scheme [10]. As part of the ENERGY-project a cross-sectional survey among more than 7000 children, their parents, and schools was conducted in seven countries representing different regions of Europe. This survey used questionnaires among children, parents, and school staff, as well as observations in the school and school environments [11].

However, for the survey no established valid and reliable measures that could be administered in large populations in different countries across Europe were available. Therefore, we developed a child and parent questionnaire to assess a range of EBRBs and potential individual and environmental behavioural determinants, and examined the test-retest reliability and construct validity of these two main questionnaires used in the ENERGY cross-sectional survey. The results of the parent questionnaire reliability and validity study are published in a separate paper [*Singh *et al*: Test-retest reliability and construct validity of the ENERGY-parent questionnaire on parenting practices, energy balance-related behaviours and their potential behavioural determinants: the ENERGY-project. submitted for publication*]. In the current paper, the methods and results of the child questionnaire test-retest reliability and construct validity study are presented and discussed.

## Methods

### Energy-child questionnaire

The ENERGY-child questionnaire was developed in order to assess EBRBs of the child as well as personal, and family and school-environmental determinants related to these EBRBs. The questionnaire was divided in eight sections, i.e. (A) Demographic characteristics; (B) Soft drinks and spending pocket money on soft drinks; (C) Fruit juices; (D) Breakfast behaviour; (E) Physical activity behaviour; (F) Screen viewing behaviour; and (G) Dieting behaviour. In the current study we assessed the test-retest reliability and construct validity of all sections (150 items), except 'demographic characteristics'.

Most concepts were measured by only one or two items due to practical constraints with regard to the length of the questionnaire. The questionnaire was developed from existing measures or such existing measures were adapted for the behaviours included in the ENERGY-child questionnaire [12-14]. More details on the development of the questionnaire, the pre-testing, and translation procedures are described elsewhere [11]. The ENERGY-child questionnaire is available via the ENERGY-website in English and all languages in which the questionnaire was administered: http://www.projectenergy.eu.

### Study population: recruitment and data collection

In the current paper, the data of the test-retest reliability and construct validity study from six out of seven countries that participated in the cross-sectional study of the ENERGY-project [10] (i.e. Belgium, Greece, Hungary, the Netherlands, Norway, and Spain) are presented. Due to deviations from the study protocol Slovenian data were excluded from the current study. Data collection, data cleaning, and data analyses were performed according to a standardized protocol and are described hereafter.

We recruited children aged 10-12 years old. The recruitment and data collection took place from March-July 2010. Children were recruited in five phases: (1) we called schools and after a short explanation of the study we asked if the school was interested in participation in the study. (2) If the school showed interest, a letter with more information on the background, goals, and methods of the study was sent. (3) A second phone call followed after one week. During this phone call, the dates on which the measurements would take place, were agreed upon. Schools were asked to select one class of children aged 10-12 years to participate in the study. (4) A second letter or email was sent to the school to confirm the dates. The letter also contained practical information on the measurements. (5) We provided schools with an information letter, which was sent to the parents of the children of the selected class. This letter contained an active/passive informed consent and detailed information on the background, goals, and methods of the study.

In countries where ethical approval was necessary for such non-intervention studies this was obtained from the relevant ethical committee and informed consent of the child and/or parents was obtained prior to the study; in the other countries a declaration of 'No objection' was obtained from the ethical committees. In Greece, both the Ministry of Education and the ethical committee approved the study protocol.

#### Test-retest reliability study

We visited the school and children were asked to fill in the ENERGY-child questionnaire in the classroom under the supervision of the researcher/research assistant. Exactly one week later, the researcher/research assistant returned and the children were asked to fill in the questionnaire for a second time. We planned the second measurement at the same part of the day as the first measurement (e.g. morning or afternoon). We collected data by ID number to be able to merge the questionnaires from the test and re-test.

#### Construct validity study

For the construct validity study a cognitive interview was conducted among approximately three children of each participating class. Before the study started, we asked the teacher to select three children representative for the class. These children were asked to volunteer for a cognitive interview with the researcher/research assistant about the same subjects as the questionnaire.

Children who participated in the construct validity study were asked to fill in the ENERGY-child questionnaire together with the other children in the class (first measurement of the test-retest reliability study) and were subsequently interviewed by a researcher/research assistant. The interview was performed using a standard question route - considering the course of the child's day from getting up until going to sleep. The interviews were sound-recorded and transcribed. Based on the transcribed interview, a second researcher/research assistant (i.e. other than the one doing the interview) filled in a second identical child questionnaire without knowledge of the answers to the first questionnaire of the children. Data of children that participated in the construct validity study were excluded from the test-retest reliability study.

### Data management

A standard data management protocol was developed to ensure missing and ambiguous values were handled consistently.

#### Double data entry

For both the test-retest reliability study and the validity study a randomly selected 5% of the questionnaires were re-entered in SPSS (double data entry) to check for typing errors and misinterpretation. A difference of less than 3% was accepted. In case there was a difference of more than 3%, the cases had to be re-entered in the original data set and the procedure was repeated. Across the countries, the rate of disagreement in the test-retest reliability and construct validity studies ranged from 0.0% - 1.7% and 0.0% - 2.3%, respectively.

#### Data definition

The data definition process consisted of adding variable labels, value labels and missing value definitions to the original data files.

#### Data cleaning

During the data cleaning original data was checked for duplicate records, system-missing values, out-of-range values and logical inconsistencies.

### Statistical analyses

#### Descriptives

We calculated means and standard deviations for the participant characteristics and medians, 25^th^, and 75^th ^percentiles values for the EBRBs.

#### Test-retest reliability and construct validity

For both test-retest reliability and construct validity we assessed agreement at the individual item level. The agreement of categorical items (mostly Likert-type scales), continuous, and dichotomous items was analysed with a two-way random effects single measure intraclass correlation coefficient (ICC 2.1); ICCs were classified as follows: 'excellent' (≥ .81), 'good' (.61 - .80), 'moderate' (.41 - .60), 'poor' (≤ .40) [3,15-17].

Because the calculation of the ICC depends on the existence of the variability in answering categories, we also calculated percentage agreement, with criteria established as 'excellent' (90% - 100%), 'good' (75% - 89%), 'moderate' (60%-74%), or 'poor' (< 60%). If ICC values were lower than .40/.60/.80 but the percentage agreement was higher than 60%/75%/90%, we reported the percentage agreement [18].

Gender-specific analyses did not show meaningful differences between boys and girls, both in the test-retest reliability and the construct validity study. Therefore, results are presented for both boys and girls combined.

All statistical tests were performed using SPSS version 15.0 (SPSS Inc., Chicago, IL).

## Results

### General

The characteristics of the children that participated in the test-retest reliability and construct validity study are shown in table 1.

**Table 1:** Descriptive statistics of the children that participated in the test-retest reliability and construct validity study.

	Belgium	Greece	Hungary	Netherlands	Norway	Spain
						
TEST-RETEST RELIABILITY STUDY*						
No. in reliability study	118	155	132	137	102	86
Age, years (mean (sd))	11.4 (.6)	11.6 (.6)	12.5 (.6)	11.8 (.7)	11.4 (.6)	11.3 (.5)
Male gender (%)	44	52	50	53	42	44
Native language at home						
Native	116	151	128	130	92	76
Non-native	2	4	1	7	10	9
Family status						
Traditional (mother and father)	99	125	88	104	85	71
Non-traditional	19	28	44	33	15	14
						
CONSTRUCT VALIDITY STUDY**						
No. in validity study	15	16	15	20	15	15
Age, years (mean (sd))	11.4 (.6)	11.5 (.6)	12.0 (.6)	11.7 (.7)	11.6 (.8)	11.6 (.8)
Male gender (%)	33	69	40	50	53	47
Native language at home						
Native	13	16	15	20	13	15
Non-native	1	0	0	0	2	0
Family status						
Traditional (mother and father)	12	14	11	13	10	13
Non-traditional	3	2	4	7	5	2

* Missing data test-retest reliability study: native language at home: n = 4 (Hungary: n = 3; Spain: n = 1); family status n = 3 (Norway: n = 2; Spain: n = 1)

** Missing data construct validity study: native language at home: n = 1 (Belgium: n = 1)

Completion of the 157-item questionnaire took about 30-60 minutes. The cognitive interviews took 35-60 minutes.

#### Test-retest reliability

There were 793 children who filled in the questionnaire for the first time. At the retest, 63 did not fill in the questionnaire and were therefore excluded from the current analysis (dropout rate: 7.9%).

In this study, we included test-retest reliability data from 730 children across the six countries. The number of participants ranged from 86 (Spain) to 155 (Greece). The mean age (standard deviation (sd)) of the children participating in the test-retest reliability study ranged from 11.3 (.5) years (Spain) to 12.5 (.6) years (Hungary). The majority of the children reported to speak the native language of the country at home.

#### Construct validity

There were 98 children who filled in the questionnaire. Two children did not show up at the interview and were therefore excluded from the current analysis (dropout rate: 3.0%).

In this study, we included construct validity data from 96 children across the six countries. All but two countries included 15 children; Greece included 16, and the Netherlands 20 children. The mean age (standard deviation (sd)) of the children participating in the test-retest reliability study ranged from 11.4 (.6) years (Belgium) to 12.0 (.6) years (Hungary). In Belgium, the majority of the children participating in the construct validity study were girls (67%), whereas in Greece the majority (69%) of the children were boys. In all countries, most children reported to speak the native language of the country at home.

#### Energy balance-related behaviours (EBRBs)

Table 2 presents the descriptives of the EBRBs, as assessed by the first completion of the questionnaire.

**Table 2:** Energy balance-related behaviours of children participating in the test-retest reliability and construct validity study. All values are medians (25^th ^- 75^th ^percentile.)

	All	Belgium	Greece	Hungary	Netherlands	Norway	Spain
**TEST-RETEST RELIABILITY STUDY**	n = 730	n = 118	n = 155	n = 132	n = 137	n = 102	n = 86
Soft drink consumption (ml/day)	154 (36-570)	214 (58-500)	36 (18-83)	580 (193-1250)	500 (89-884)	130 (36-392)	141 (41-463)
Fruit juice consumption (ml/day)	196 (41-356)	158 (36-268)	141 (36-250)	249 (83-497)	249 (107-580)	107 (18-214)	231 (41-456)
Breakfast consumption (days/week)	7.0 (6.0-7.0)	7.0 (6.0-7.0)	7.0 (5.0-7.0)	6.0 (3.0-7.0)	7.0 (7.0-7.0)	7.0 (6.0-7.0)	7.0 (7.0-7.0)
Cycling (min/day)	.0 (.0-6.0)	6.0 (.0-16.0)	.0 (.0-.0)	.0 (.0-6.0)	6.0 (.0-16.0)	6.0 (6.0-6.0)	.0 (.0-3.0)
Walking (min/day)	6.0 (.0-16.0)	6.0 (.0-16.0)	6.0 (6.0-16.0)	6.0 (.0-26.0)	6.0 (.0-16.0)	16.0 (6.0-26.0)	6.0 (.0-16.0)
Sport (min/day)	30.0 (17.1-42.9)	34.3 (17.1-45.0)	30.0 (17.1-42.9)	30.0 (17.1-42.9)	30.0 (17.1-42.9)	30.0 (17.1-51.4)	25.7 (17.1-51.4)
Watching TV (min/day)	107.1 (68.6-150.0)	102.9 (68.6-136.1)	113.7 (77.1-158.6)	120.0 (81.4-182.1)	98.6 (68.6-141.4)	77.1 (49.3-126.4)	98.6 (64.3-154.3)
Computer usage (min/day)	68.6 (34.3-115.7)	64.3 (30.0-98.6)	68.6 (38.6-111.4)	98.6 (51.4-158.6)	81.4 (42.9-135.0)	60.0 (30.0-94.3)	51.4 (30.0-90.0)
							
**CONSTRUCT VALIDITY STUDY**	n = 96	n = 15	n = 16	n = 15	n = 20	n = 15	n = 15
Soft drink consumption (ml/day)	107 (36-384)	36 (0-249)	36 (19-133)	107 (36-589)	428 (116-962)	154 (71-214)	77 (36-214)
Fruit juice consumption (ml/day)	196 (36-393)	107 (0-250)	231 (107-250)	214 (36-463)	249 (36-830)	143 (18-393)	107 (36-250)
Breakfast consumption (days/week)	7.0 (6.0-7.0)	7.0 (6.8-7.0)	7.0 (5.3-7.0)	5.0 (4.0-7.0)	7.0 (6.0-7.0)	7.0 (6.0-7.0)	7.0 (6.8-7.0)
Cycling (min/day)	.0 (.0-6.0)	6.0 (.0-6.0)	.0 (.0-.0)	.0 (.0-6.0)	6.0 (1.5-16.0)	16.0 (6.0-16.0)	.0 (.0-.0)
Walking (min/day)	6.0 (1.5-26.0)	6.0 (.0-26.0)	6.0 (1.5-16.0	16.0 (.0-26.0)	6.0 (.0-16.0)	26.0 (6.0-40.0)	6.0 (.0-16.0)
Sport (min/day)	34.3 (20.4-55.7)	45.0 (24.6-62.1)	38.6 (27.9-51.4)	48.9 (27.9-55.7)	27.9 (20.4-51.4)	23.6 (16.1-60.0)	19.3 (8.6-42.9)
Watching TV (min/day)	111.4 (68.6-154.3)	77.1 (68.6-120.0)	124.3 (91.1-160.7)	115.7 (68.6-158.6)	126.4 (79.6-157.5)	115.7 (68.6-167.1)	92.1(60.0-123.2)
Computer usage (min/day)	68.6 (30.0-120.0)	68.6 (30.0-120.0)	68.6 (38.6-128.6)	72.9 (60.0-175.7)	75.0 (51.4-124.3)	57.9 (24.6-97.5)	42.9 (33.2-64.3)

### General findings test-retest reliability and construct validity study

Table 3 shows the questionnaire items, their ICC values, and percentage agreement for all countries combined, for both the test-retest reliability and construct validity study. Table 4 summarises these findings per category of the ENERGY-child questionnaire.

**Table 3:** Agreement (per questionnaire item) between questionnaires (test-retest reliability) and questionnaire and interview responses (construct validity) as indicated by intraclass correlation coefficients (ICC) and percentage agreement (agree).

Item	reliability		validity	
	**ICC**	**agree**	**ICC**	**agree**

How many times a week do you usually drink fizzy drinks and fruit squash?	.71	55	.59	55

On a day that you drink fizzy drinks and fruit squash, how many glasses, cans or bottles do you drink on such a day? *Glasses or small bottles (250 ml)*	.59	55	.24	58

On a day that you drink fizzy drinks and fruit squash, how many glasses, cans or bottles do you drink on such a day? *Cans (330 ml)*	.53	73	.44	71

On a day that you drink fizzy drinks and fruit squash, how many glasses, cans or bottles do you drink on such a day? *Bottles (500 ml)*	.58	77	-.01	81

How many fizzy drinks or fruit squash did you drink yesterday? *Glasses or small bottles (250 ml)*	.58	88	.48	64

How many fizzy drinks or fruit squash did you drink yesterday? *Cans (330 ml)*	.53	82	.21	86

How many fizzy drinks or fruit squash did you drink yesterday? *Bottles (500 ml)*	.55	84	.10	92

I think that drinking fizzy drinks or fruit squash is......	.68	65	.28	38

I think drinking fizzy drinks or fruit squash will make me fat	.55	49	.42	45

If I drink fizzy drinks or fruit squash, my parents/care givers think this is......	.53	66	.51	51

If I drink fizzy drinks or fruit squash, most of my friends think this is......	.63	65	.40	48

How often do your parents/care givers drink fizzy drinks or fruit squash?	1.00	60	1.00	51

How often do most of your friends drink fizzy drinks or fruit squash?	.57	62	.42	60

I like the taste of fizzy drinks or fruit squash	.67	68	.32	58

Drinking fizzy drinks or fruit squash is something that I do without even really thinking about	.59	47	.25	34

I find drinking no fizzy drinks or fruit squash...	.59	55	.22	32

If I ask my parents/care givers for a fizzy drink or fruit squash, I get one	.62	54	.54	44

I am allowed to take fizzy drinks or fruit squash whenever I want	.68	52	.30	43

Do your parents/care givers have rules about how many fizzy drinks or fruit squash you are allowed to drink?	.60	80	.49	75

If you ask your parents/care givers to buy a certain brand of fizzy drinks or fruit squash, will she do it?	.65	55	.42	33

Are there usually fizzy drinks or fruit squash at your home?	.74	61	.52	46

In which situations do you usually drink fizzy drinks or fruit squash? *During the weekend*	.54	80	.31	63

In which situations do you usually drink fizzy drinks or fruit squash? *Breakfast*	.42	94	-.01	98

In which situations do you usually drink fizzy drinks or fruit squash? *Lunch*	.54	83	.32	88

In which situations do you usually drink fizzy drinks or fruit squash? *Dinner*	.59	85	.30	80

In which situations do you usually drink fizzy drinks or fruit squash? *At school*	.64	91	.19	92

In which situations do you usually drink fizzy drinks or fruit squash? *While watching television*	.56	81	.35	81

In which situations do you usually drink fizzy drinks or fruit squash? *As a thirst quencher between meals*	.50	82	.27	86

In which situations do you usually drink fizzy drinks or fruit squash? *During/after sports*	.60	84	.36	80

In which situations do you usually drink fizzy drinks or fruit squash? *When I am with friends*	.48	74	.28	65

In which situations do you usually drink fizzy drinks or fruit squash? *At birthdays/parties*	.45	83	.27	72

In which situations do you usually drink fizzy drinks or fruit squash? *I never drink fizzy drinks or fruit squash*	.56	97	.32	95

How often do you spend your own money on fizzy drinks or fruit squash?	.65	67	.32	53

If the price of fizzy drinks and fruit squash were doubled, I would buy less fizzy drinks or fruit squash from my own money	.61	56	.42	42

How many times a week do you usually drink fruit juices?	.64	48	.69	52

On a day that you drink fruit juices, how many glasses or cartons do you drink on such a day? *Glasses or small cartons (250 ml)*	.54	60	.54	51

On a day that you drink fruit juices, how many glasses or cartons do you drink on such a day? *Regular cartons (330 ml)*	.52	73	.20	78

How many fruit juices did you drink yesterday? *Glasses or small cartons (250 ml)*	.48	53	.65	63

How many fruit juices did you drink yesterday? *Regular cartons (330 ml)*	.35	84	.17	83

I think that drinking fruit juices is...	.62	65	.19	50

I think it is recommended for children my age...	.54	67	.39	51

I think drinking fruit juices will make me fat.	.45	54	.45	47

I am allowed to take fruit juices whenever I want	.67	59	.48	53

Do your parents/care givers have rules about how many fruit juices you are allowed to drink?	.64	86	.68	87

Are there usually fruit juices in your home?	.67	59	.57	52

In which situations are you most likely to drink fruit juices? *During the weekend*	.41	70	.30	71

In which situations are you most likely to drink fruit juices? *Breakfast*	.57	78	.57	79

In which situations are you most likely to drink fruit juices? *Lunch*	.44	77	.24	73

In which situations are you most likely to drink fruit juices? *Dinner*	.41	81	.32	81

In which situations are you most likely to drink fruit juices? *At school*	.57	82	.29	87

In which situations are you most likely to drink fruit juices? *While watching television*	.47	81	.25	84

In which situations are you most likely to drink fruit juices? *As a thirst quencher between meals*	.46	76	.04	70

In which situations are you most likely to drink fruit juices? *During/after sports*	.46	77	.14	77

In which situations are you most likely to drink fruit juices? *When I am with friends*	.41	76	.62	88

In which situations are you most likely to drink fruit juices? *At birthdays/parties*	.42	71	.45	76

In which situations are you most likely to drink fruit juices? *I never drink fruit juices*	.53	98	.85	99

From Monday to Friday during school weeks, on how many days do you usually eat breakfast?	.73	83	.23	80

On how many days in the weekend days (Saturday and Sunday) do you usually eat breakfast?	.52	81	.39	87

What do you usually have for breakfast on school days?	.57	65	.63	71

What is the reason that you usually skip breakfast?	.74	83	.58	76

Did you eat breakfast yesterday?	.64	91	.26	91

Did you eat lunch yesterday?	.33	94	.00	96

Did you eat dinner yesterday?	.33	92	1.00	100

Did you eat anything between meals yesterday?	.42	76	.43	79

I think that eating breakfast is...	.59	77	.01	44

I think it is recommended for children of my age to...	.39	75	.30	77

I think not eating breakfast will make me fat	.44	54	.45	51

I think eating breakfast will make me fat	.48	55	.36	29

If I eat breakfast, my parents/care givers think this is...	.53	77	.21	52

If I eat breakfast, most of my friends think this is...	.53	63	.38	54

How often do your parents/care givers eat breakfast?	.71	72	.69	73

How often do most of your friends eat breakfast?	.53	63	.34	44

I like eating breakfast	.65	72	.29	57

Eating breakfast is something that I do without even really thinking about it	.59	54	.15	41

I find eating breakfast every day...	.71	65	.36	50

My parents/care givers encourage me to have breakfast	.65	59	.40	54

Do your parents/care givers have rules about whether you should eat breakfast?	.55	79	.35	67

If you ask your parents/care givers to buy a certain brand of food or drink for breakfast, will they do it?	.53	56	.34	51

Are there usually breakfast products (milk, cereals, bread et cetera) at your home?	.42	75	.25	80

How often do you eat breakfast with your parents/care givers?	.74	51	.66	41

In which situations do you usually eat your breakfast? *At a set table at home*	.67	91	.16	86

In which situations do you usually eat your breakfast? *In bed*	.56	95	.66	98

In which situations do you usually eat your breakfast? *While watching television*	.62	85	.36	84

In which situations do you usually eat your breakfast? *On my way to school*	.43	96	.00	98

In which situations do you usually eat your breakfast? *At school before the class starts*	.44	94	-.02	96

In which situations do you usually eat your breakfast? *I never eat breakfast*	.45	96	.00	99

How many days do you usually bike to school?	.94	88	.81	73

If you bike to school, how long does it take you to bike to school?	.81	85	.66	75

How many days a week do you usually walk to school?	.91	81	.84	75

If you walk to school, how long does it take you to walk to school?	.70	76	.59	74

How many days do you usually travel by car to school?	.91	84	.84	80

How many days do you usually travel by public transport (bus, school bus, tram, metro) to school?	.88	92	.81	96

How did you go to school today?	.79	83	.67	74

What do you usually do during breaks at school?	.80	86	.65	81

I do not participate in any sports activities	.76	92	.84	95

In a total week how many hours do you do this sport?	.74	55	.61	50

I do not have a second sport	.68	84	.51	75

In a total week how many hours do you do this sport?	1.00	43	1.00	36

How many hours of sports did you do yesterday?	.22	28	.22	50

I think that physical activity/sports is......	.54	86	.04	68

I think it is recommended for children of my age......	.47	48	.09	39

I think NOT doing physical activity/sports will make me fat	.49	54	.47	55

If I do physical activity/sports, my parents/care givers think this is......	.46	81	-.02	51

If I do physical activity/sports, most of my friends think this is......	.58	71	.35	58

How often do your parents/care givers do physical activity/sports?	.67	58	.59	47

How often do most of your friends do physical activity/sports?	.55	66	.32	55

I like doing physical activity/sports	.64	81	.09	80

Physical activity/sports is something that I do without even really thinking about it.	.56	52	.08	32

I find doing physical activity/sports for 1 hour every day......	.64	71	.15	37

My parents/care givers encourage me to be physically active/do sports	.65	62	.37	52

My parents/care givers help me if I need something for my sports	.58	76	.39	80

Do your parents/care givers have rules about whether you should be physically active/do sports?	.46	77	.49	77

Do your parents/care givers allow you to take part in physical activity/do sports?	.21	94	1.00	100

If you indicate that you like a certain physical activity/sports will your parents/care givers allow you to do it?	.62	63	.27	50

Do you have the following things at home that you can use for physical activities/sports? *Bike*	.54	91	.64	91

Do you have the following things at home that you can use for physical activities/sports? *Tennis and or badminton racket*	.66	85	.34	66

Do you have the following things at home that you can use for physical activities/sports? *Ball*	.49	91	.20	80

Do you have the following things at home that you can use for physical activities/sports? *Sporting shoes*	.49	89	.06	54

Do you have the following things at home that you can use for physical activities/sports? *Skipping rope*	.75	89	.42	71

Do you have the following things at home that you can use for physical activities/sports? *Skates*	.78	90	.39	69

Do you have the following things at home that you can use for physical activities/sports? *Skis*	.81	93	.49	83

Do you have the following things at home that you can use for physical activities/sports? *Skate board*	.76	88	.45	74

How often do you take part in physical activity/do sports with your parents/care givers?	.47	47	.24	51

About how many hours a day do you usually watch television in your free time? Week days (average of all weekdays)	.67	42	.63	44

About how many hours a day do you usually watch television in your free time? Weekend days (average of all weekend days)	.68	36	.56	40

About how many hours a day do you usually play games on a computer, or use your computer for leisure activities in your free time? Week days (average of all week days)	.67	41	.35	30

About how many hours a day do you usually play games on a computer, or use your computer for leisure activities in your free time? Weekend days (average of all weekend days)	.67	38	.65	37

About how many hours did you watch television yesterday?	.68	36	.70	44

About how many hours did you play games on a computer, games console or use your computer for leisure activities yesterday?	.54	39	.28	51

I think watching television is...	.68	66	.19	44

I think it is recommended for children of my age...	.48	46	.26	30

I think watching too much television can help making me fat	.65	56	.55	42

If I watch television, my parents/care givers think this is...	.52	69	.23	54

If I watch television, most of my friends think this is...	.64	67	.52	49

How often do your parents/care givers watch television?	.68	63	.34	47

How often do most of your friends watch television?	.49	62	.28	53

I like watching television	.67	63	.45	50

Watching television is something that I do without even really thinking about	.65	55	.30	34

I find not watching television...	.64	53	.43	30

My parents/care givers allow me to watch television whenever I want	.68	51	.35	33

If I ask my parents/care givers to watch television, I can do so	.63	61	.39	57

Do your parents/care givers have rules about how many hours per day you are allowed to watch television?	.60	80	.40	68

Do you have a television in your own bedroom?	.92	96	.81	91

How often do you watch television with your parents/care givers?	.64	41	.49	37

How often do you watch television during meals? *Breakfast*	.76	69	.66	64

How often do you watch television during meals? *Lunch*	.75	60	.67	58

How often do you watch television during meals? *Dinner*	.77	57	.61	46

Do you think you are too thin or too fat?	.87	83	.68	74

How often have you tried to get slimmer/thinner during the last year?	.79	77	.65	75

Do you try to get slimmer or thinner right now?	.72	89	.60	84

Results are presented for all countries combined.

**Table 4:** Overview of the results per section of the ENERGY child-questionnaire for both test-retest reliability and construct validity study, combined for all countries (test-retest reliability study: n = 730; construct validity study: n = 96).

		test-retest reliability					construct validity				
**section of the ENERGY-child questionnaire**	**# items**	**range**	**excellent** **n (%)**	**good** **n (%)**	**moderate** **n (%)**	**poor** **n (%)**	**range**	**excellent** **n (%)**	**good** **n (%)**	**moderate** **n (%)**	**poor** **n (%)**
soft drinks	34	.42 - 1.00	4 (11.8)	22 (64.7)	8 (23.5)	-	.10 - 1.00	5 (14.7)	7 (20.6)	14 (41.2)	8 (23.5)
fruit juices	22	.35 - .67	1 (4.5)	14 (63.6)	7 (31.8)	-	.04 - .85	1 (4.5)	12 (54.5)	7 (31.8)	2 (9.1)
breakfast	30	.33 - .74	8 (26.7)	13 (43.3)	9 (30.0)	-	.00 - 1.00	7 (23.3)	11 (36.7)	2 (6.7)	10 (33.3)
physical activity behaviour	37	.21 - 1.00	11 (29.7)	19 (51.4)	6 (16.2)	1 (2.7)	.04 - 1.00	7 (18.9)	10 (27.0)	9 (24.3)	11 (29.7)
screen viewing behaviour	24	.48 - .92	1 (4.2)	19 (76.2)	4 (16.7)	-	.23 - .81	2 (8.3)	5 (20.8)	7 (29.2)	10 (41.7)
dieting behaviour	3	.72 - .87	1 (33.3)	2 (66.7)	-	-	.60 - .68	-	3 (100)	-	-

**Overall**	150	.21 - 1.00	26 (17.3)	89 (59.3)	34 (22.7)	1 (.7)	.00 - 1.00	22 (14.7)	48 (32.0)	39 (26.0)	41 (27.3)

#### Test-retest reliability study

For the total sample across all countries, the test-retest reliability was good to excellent in 115 (76.6%) items and moderate in 34 (22.7%) items. For one item ('*How many hours of sports did you do yesterday?*') we found an ICC-value of .22, indicating poor test-retest reliability. Eleven response items did not show enough variability, resulting in ICCs ≤ .60, but a high (≥90%) percentage agreement (table 3). The test-retest reliability was comparable across all countries. Country-specific values can be found in additional file 1.

#### Construct validity study

Construct validity appeared to be good to excellent for 70 out of 150 items (46.7%), as indicated by ICCs > .60 or percentage agreement ≥ 75%. For the remaining part, the ICCs of 39 items (26.0%) indicated moderate construct validity and 41 items (27.3%) indicated poor construct validity.

Constructs that showed consistently poor values across most of the EBRBs were

- general attitude (e.g. '*I think watching television is*....')

- automaticity (e.g. '*Drinking fizzy drinks or fruit squash is something I do without even really thinking abou*t')

- parental and peer subjective norm ('*If I watch television my parents/care givers think it is...' *or '*If I do physical activity/sports, most of my friends think it is*...')

Nine response items did not show enough variability, resulting in ICCs ≤ .40, but high (≥ 90%) percentage agreement (table 3).

The construct validity was comparable across all countries, except for Greece and the Netherlands. Greek data showed higher ICCs and percentages agreement (see additional file 2). The construct validity in Greece was excellent in about two thirds (68.0%) of the items, good in 19.3%, moderate in 10.7%, and poor in 2.0% of the items. Dutch data showed lower ICCs and percentages agreement. The construct validity in the Netherlands was excellent to good in 46.7% of the items, moderate in 26.0%, and poor in 27.3% of the items.

## Discussion

The current study assessed the test-retest reliability and construct validity of the ENERGY-child questionnaire in 10-12 year old children from six countries in Europe. The ENERGY-child questionnaire, assessing EBRBs of the child as well as potential personal, family, and school-environmental correlates of these EBRBs, showed good test-retest reliability and moderate to good construct validity.

In the light of the scarcity of the published reliable and valid instruments that simultaneously assess both sides of the energy balance, the results of the current study should be helpful for future research.

### Test-retest reliability

More than three quarter of all items (n = 115 out of 150) of the ENERGY-child questionnaire showed good to excellent test-retest reliability.

Exceptions on these findings are the questions in which children were asked about 'yesterday' (e.g. '*About how many hours did you watch television yesterday*?'). Here we find lower values especially on items like consumption of soft drinks, television watching and sports participation. Lower ICCs or percentage agreement are to be expected regarding such a question, because children will have larger variety in activities engaged in yesterday compared to on a usual day.

With comparable results across all countries, our results show that the ENERGY-child questionnaire has good test-retest reliability in the six European countries that participated in the current study.

### Construct validity

Values for the construct validity were somewhat lower than those for the test-rest reliability. A closer examination of the questions showed that across all EBRBs, several constructs had consistently lower scores (i.e. general attitude, habit strength, parental and peer subjective norm). The lower validity for the habit strength questions is consistent with the findings of the ENERGY-parent questionnaire, where we also found lower values for the habit strength questions [Singh *et al*: Test-retest reliability and construct validity of the ENERGY-parent questionnaire on parenting practices, energy balance-related behaviours and their potential behavioural determinants: the ENERGY-project. submitted for publication]. These findings indicate that the use of single questions out of the original habit strength index [19] is not to be advised, and other habit strength questionnaire items should be considered in future research.

All items that have poor values should be reconsidered and in interpreting research results based on these items the lack of construct validity should be noted.

Some differences between countries were observed, i.e. Greece and the Netherlands, but because the number of cases per country was relatively small (Greece: n = 16 and the Netherlands: n = 20), we believe that more value should be attached to the combined data set.

However, for future interpretation of results of the ENERGY-study, these country-specific values might be helpful, explaining cross-country differences.

### Comparison with other studies

Only few studies have reported on the psychometric properties of child questionnaires assessing a range of EBRBs. The psychometric properties of the Health Behaviours in School Children (HBSC) questionnaire has been reported in two different papers [20,21]. Vereecken *et al*. [20] reported on the reliability and validity of questionnaire items aiming to assess a number of food items from the HBSC-questionnaire (HBSC FFQ) - among which soft drinks. Test-rest reliability for soft drink consumption was comparable to the values we found: in 11-12 year olds, Vereecken *et al*. [20] report a weighed kappa of .66 (percentage agreement: 53%). Similar to our results, the score for validity was somewhat lower. It is noteworthy, that Vereecken *et al*. [20] mention that overestimation is very likely when measuring food items such as soft drinks.

Booth *et al*. [21] assessed the reliability and validity of the physical activity questions of the HBSC-questionnaire among Australian adolescents. Reliability was assessed in a comparable way to our study (i.e. administered twice, two weeks apart). The concept of the questions in the HBSC-questionnaire assessing participation in (vigorous) physical activity by examining the frequency ('How often') and duration ('How long') of vigorous physical activity was clearly different from the way the ENERGY-child questionnaire assessed sports participation, i.e. 'How many hours...?'. Booth *et al*. [21] concluded that the HBSC-questions on participation in vigorous intensity physical activity had acceptable reliability, with values for children with a mean age of 13.7 years ranging between .36 - .44 (frequency) and .22 - .26 (duration).

There are at least two other studies that are worth comparing our results to, i.e. a study among children of the same age range, focusing on energy intake [22] and another cross-European study focussing on fruit and vegetable consumption [14]. Wilson *et al*. [22] examined the psychometric properties of a questionnaire among 10-12 year olds. The authors conclude that this 54-item questionnaire is to be a reliable and valid tool to assess dietary patterns and food behaviours, attitudes and environments in Australian school children [22]. Similar to the ENERGY-child questionnaire, Wilson *et al*. [22] assessed intake of sweetened beverages and found similar values for both reliability (.59) and validity (.34). The range of ICC values of the ENERGY-child questionnaire was somewhat broader than those reported by Wilson *et al*. [22]. This might be due to the fact that Wilson *et al*. present the ICCs for sum scores instead of single items, as we did. The fact that their study population for the test-retest reliability was much smaller (n = 134 versus 730) and that they examined a questionnaire focusing on energy intake and its determinants prohibits further comparison.

Comparing our study to other studies that assessed validity, it should be considered that both Vereecken *et al*. [20] and Wilson *et al*. [22] compared their questionnaires to 7-day food diaries, whereas in our study we conducted an interview assessing construct validity. Comparison to food records assesses the relative validity of the questionnaire and may be regarded as a more rigorous test of validity.

De Bourdeaudhuij *et al*. [14] examined the reliability and validity of a questionnaire assessing personal, social and environmental correlates of fruit and vegetable intake in schoolchildren in five European countries. The authors conclude that the questionnaire is a reliable and valid tool for 10-11 year-olds. Comparable to our study, they report good to very good test-retest reliability for the majority of the items. Again, detailed comparison with the results of the present validity study is not possible, because de Bourdeaudhuij *et al*. [14] examined predictive validity instead of construct validity.

### Strengths and limitations

The test-retest study has several strengths, covering both the data collection and handling phase (i.e. large sample size, standardised protocol, centralised data management) and a questionnaire covering a large variety of children's EBRBs as well as potential personal, family, and school-environmental determinants, available for administration in nine languages.

However, also some limitations should be mentioned when interpreting our results. The study sample of the construct validity study was relatively small and therefore not fully representative of the total population of children across the countries represented, limiting the generalizability of the findings of the construct validity study.

The lack of a 'gold standard' in the validation study must be considered as a major limitation; such gold standards are just not available for assessment of most EBRBs or potential behavioural determinants. We chose to investigate construct validity of the questionnaire by comparing the answers of the questionnaire to the answers given in a face-to-face interview. The method of comparing questionnaires to interviews has been previously used to validate parent questionnaires on children's physical activity correlates [23]. Using interviews also enabled us to learn whether the respondents interpreted the questions as we intended. We therefore think that the use of face-to-face interviews was a strength of the current study, adding important feedback and gaining more insight into the participants' answers. Three shortcomings of this method for the validation of the questionnaire should be mentioned. First, the interpretation of the responses in the interview might lead to bias. We attempted to minimise this bias by following a strict data entry protocol, i.e. the face-to-face interviewer was another person as the one who filled in a second questionnaire based on the interview results. A second shortcoming lies within the fact that both data, i.e. from the questionnaire and the interview, were based on self-report, making it likely that there is correlated error between both measures. A third and general shortcoming of subjective reporting is that answers are more likely to be given in a social desirable direction, and a face-to-face interview is likely to increase this bias. We aimed to minimise this form of bias by clearly indicating the importance of honest answers instead of social desirable answers before the interview.

When interpreting the results, it should be considered that in the current study protocol, data were not collected on Mondays, to make sure that the questions referring to 'yesterday' did not cover a weekend day. Most probably, recalling activities on weekend days is more difficult for children, when compared to weekdays, since the latter tend to be more structured [5].

The current study examined the test-retest reliability and construct validity of the ENERGY-child questionnaire. Internal consistency was not assessed because most constructs were assessed by only one or two items. Future studies need to establish other aspects of validity and reliability such as content validity and responsiveness.

## Conclusion

Our results demonstrate that the ENERGY-child questionnaire, assessing EBRBs of the child as well as personal, family, and school-environmental determinants related to these EBRBs, has good test-retest reliability and moderate to good construct validity.

Being able to validly and reliably assess EBRBs and several potential determinants of those EBRBs in different languages and countries will enable future observational and intervention research regarding childhood overweight and its drivers.

## Competing interests

The authors declare that they have no competing interests.

## Authors' contributions

ASS, MJMC, MM and JB developed the measurement instrument. ASS developed the study protocol, coordinated and supervised the international data collection. ASS, FNV, MV, JMFA, SS and YM contributed to or supervised the national data collection procedures. LU conducted the data analyses under supervision of ASS and MJMC. FNV conducted the data analyses under supervision of ASS and LU. ASS drafted the manuscript. All authors read and approved the final manuscript.

### Funding

The ENERGY-project is funded by the Seventh Framework Programme (CORDIS FP7) of the European Commission, HEALTH (FP7-HEALTH-2007-B). The content of this article reflects only the authors’ views and the European Community is not liable for any use that may be made of the information contained therein. 

## Supplementary Material

Additional file 1 **Table**. Country-specific results of the test-retest reliability study of the ENERGY-child questionnaire: agreement (per questionnaire item) between questionnaires as indicated by intraclass correlation coefficients (ICC) and percentage agreement (agree). Country-specific results of the test-retest reliability study of the ENERGY-child questionnaire. Click here for file

Additional file 2 **Table**. Country-specific results of the construct validity study of the ENERGY-child questionnaire: agreement (per questionnaire item) between questionnaire and interview as indicated by intraclass correlation coefficients (ICC) and percentage agreement (agree). Click here for file
