# Supplementary material for: Test-retest reliability and construct validity of the ENERGY-child questionnaire on energy balance-related behaviours and their potential determinants: the ENERGY-project
Source: Int J Behav Nutr Phys Act. 2011 Dec 9;8:136. doi: 10.1186/1479-5868-8-136 (PMC3293727; doi:10.1186/1479-5868-8-136)
Supplement: Additional file 1 — Table. Country-specific results of the test-retest reliability study of the ENERGY-child questionnaire: agreement (per questionnaire item) between questionnaires as indicated by intraclass correlation coefficients (ICC) and percentage agreement (agree). Country-specific results of the test-retest reliability study of the ENERGY-child questionnaire. [file 1479-5868-8-136-S1.DOC]

**Additional file 1.**

**Table.** Country-specific results of the test-retest reliability study of the ENERGY-child questionnaire: agreement (per questionnaire item) between questionnaires as indicated by intraclass correlation coefficients (ICC) and percentage agreement (agree).

| item/country | Belgium | | Greece | | Hungary | | Netherlands | | Norway | | Spain | |
| --- | --- | --- | --- | --- | --- | --- | --- | --- | --- | --- | --- | --- |
|  | ICC | agree | ICC | agree | ICC | agree | ICC | agree | ICC | agree | ICC | agree |
| How many times a week do you usually drink fizzy drinks and fruit squash? | .69 | 59 | .61 | 57 | .51 | 42 | .75 | 55 | .73 | 59 | .67 | 60 |
| On a day that you drink fizzy drinks and fruit squash, how many glasses, cans or bottles do you drink on such a day? Glasses or small bottles (250 ml) | .66 | 59 | .48 | 73 | .19 | 30 | .70 | 55 | .55 | 52 | .62 | 59 |
| On a day that you drink fizzy drinks and fruit squash, how many glasses, cans or bottles do you drink on such a day? Cans (330 ml) | .54 | 74 | .40 | 75 | .53 | 66 | .56 | 71 | .63 | 78 | .39 | 73 |
| On a day that you drink fizzy drinks and fruit squash, how many glasses, cans or bottles do you drink on such a day? Bottles (500 ml) | .47 | 74 | -.02 | 94 | .49 | 64 | .52 | 74 | .41 | 71 | .43 | 77 |
| How many fizzy drinks or fruit squash did you drink yesterday? Glasses or small bottles (250 ml) | .55 | 53 | .20 | 70 | .42 | 39 | .66 | 72 | .31 | 57 | .34 | 63 |
| How many fizzy drinks or fruit squash did you drink yesterday? Cans (330 ml) | .63 | 90 | .38 | 86 | .62 | 72 | .57 | 82 | .17 | 83 | .15 | 80 |
| How many fizzy drinks or fruit squash did you drink yesterday? Bottles (500 ml) | .30 | 84 | -.01 | 99 | .56 | 59 | .49 | 83 | .04 | 85 | .15 | 93 |
| I think that drinking fizzy drinks or fruit squash is...... | .52 | 63 | .47 | 53 | .64 | 59 | .66 | 72 | .58 | 68 | .56 | 66 |
| I think drinking fizzy drinks or fruit squash will make me fat | .61 | 46 | .56 | 56 | .51 | 47 | .65 | 55 | .45 | 43 | .32 | 42 |
| If I drink fizzy drinks or fruit squash, my parents/care givers think this is...... | .60 | 75 | .35 | 49 | .50 | 65 | .62 | 71 | .45 | 64 | .38 | 65 |
| If I drink fizzy drinks or fruit squash, most of my friends think this is...... | .45 | 61 | .53 | 59 | .60 | 72 | .50 | 72 | .56 | 74 | .50 | 51 |
| How often do your parents/care givers drink fizzy drinks or fruit squash? | 1.00 | 65 | 1.00 | 55 | 1.00 | 52 | 1.00 | 62 | 1.00 | 67 | 1.00 | 43 |
| How often do most of your friends drink fizzy drinks or fruit squash? | .50 | 69 | .43 | 55 | .55 | 66 | .57 | 63 | .51 | 67 | .31 | 50 |
| (B10) I like the taste of fizzy drinks or fruit squash. | .76 | 78 | .63 | 60 | .48 | 59 | .72 | 75 | .76 | 82 | .57 | 50 |
| Drinking fizzy drinks or fruit squash is something that I do without even really thinking about. | .57 | 50 | .58 | 43 | .64 | 50 | .57 | 50 | .58 | 43 | .46 | 45 |
| I find drinking no fizzy drinks or fruit squash..... | .71 | 56 | .47 | 54 | .57 | 51 | .63 | 58 | .62 | 57 | .32 | 24 |
| If I ask my parents/care givers for a fizzy drink or fruit squash, I get one. | .67 | 62 | .57 | 49 | .54 | 50 | .54 | 53 | .59 | 59 | .39 | 52 |
| I am allowed to take fizzy drinks or fruit squash whenever I want. | .70 | 56 | .60 | 52 | .61 | 50 | .69 | 53 | .64 | 46 | .49 | 34 |
| Do your parents/care givers have rules about how many fizzy drinks or fruit squash you are allowed to drink? | .63 | 81 | .50 | 79 | .58 | 80 | .62 | 82 | .58 | 79 | .58 | 80 |
| If you ask your parents/care givers to buy a certain brand of fizzy drinks or fruit squash, will she do it? | .72 | 60 | .66 | 50 | .62 | 57 | .64 | 55 | .63 | 63 | .30 | 85 |
| Are there usually fizzy drinks or fruit squash at your home? | .72 | 67 | .70 | 57 | .72 | 46 | .74 | 64 | .71 | 63 | .52 | 48 |
| In which situations do you usually drink fizzy drinks or fruit squash? *During the weekend* | .71 | 89 | .41 | 70 | .53 | 78 | .44 | 77 | .00 | 93 | .52 | 77 |
| In which situations do you usually drink fizzy drinks or fruit squash? *Breakfast* | -.01 | 98 | -.01 | 99 | .52 | 88 | .43 | 96 | .00 | 20 | .00 | 93 |
| In which situations do you usually drink fizzy drinks or fruit squash? *Lunch* | .59 | 84 | .48 | 84 | .57 | 83 | .53 | 84 | .00 | 44 | .60 | 85 |
| In which situations do you usually drink fizzy drinks or fruit squash? *Dinner* | .55 | 84 | .52 | 86 | .47 | 83 | .71 | 87 | .00 | 69 | .52 | 86 |
| In which situations do you usually drink fizzy drinks or fruit squash? *At school* | .00 | 99 | .36 | 92 | .73 | 87 | .59 | 86 | .00 | 33 | -.05 | 91 |
| In which situations do you usually drink fizzy drinks or fruit squash? *While watching television* | .58 | 83 | .19 | 86 | .51 | 78 | .54 | 77 | .00 | 50 | .68 | 90 |
| In which situations do you usually drink fizzy drinks or fruit squash? *As a thirst quencher between meals* | .39 | 81 | .51 | 92 | .55 | 79 | .38 | 72 | .00 | 50 | .55 | 90 |
| In which situations do you usually drink fizzy drinks or fruit squash? *During/after sports* | .64 | 86 | .40 | 90 | .67 | 86 | .58 | 79 | .00 | 50 | .29 | 78 |
| In which situations do you usually drink fizzy drinks or fruit squash? *When I am with friends* | .50 | 75 | .49 | 76 | .25 | 63 | .54 | 77 | .00 | 72 | .50 | 84 |
| In which situations do you usually drink fizzy drinks or fruit squash? *At birthdays/parties* | .58 | 85 | .42 | 83 | .50 | 80 | .30 | 81 | .00 | 93 | .40 | 78 |
| In which situations do you usually drink fizzy drinks or fruit squash? *I never drink fizzy drinks or fruit squash* | .50 | 98 | .24 | 96 | .00 | 99 | .72 | 96 | .00 | 33 | .66 | 98 |
| How often do you spend your own money on fizzy drinks or fruit squash? | .72 | 84 | .65 | 63 | .58 | 50 | .77 | 77 | .50 | 64 | .37 | 63 |
| (B20) If the price of fizzy drinks and fruit squash were doubled, I would buy less fizzy drinks or fruit squash from my own money. | .56 | 64 | .56 | 45 | .45 | 54 | .69 | 66 | .69 | 58 | .52 | 55 |
| How many times a week do you usually drink fruit juices? | .67 | 47 | .59 | 48 | .60 | 53 | .54 | 46 | .67 | 54 | .72 | 46 |
| On a day that you drink fruit juices, how many glasses or cartons do you drink on such a day? *Glasses or small cartons (250 ml)* | .55 | 59 | .25 | 70 | .51 | 52 | .49 | 46 | .70 | 68 | .57 | 69 |
| On a day that you drink fruit juices, how many glasses or cartons do you drink on such a day? *Regular cartons (330 ml)* | .40 | 85 | .21 | 83 | .40 | 51 | .47 | 67 | .31 | 86 | .59 | 63 |
| How many fruit juices did you drink yesterday? *Glasses or small cartons (250 ml)* | .65 | 62 | .38 | 55 | .31 | 41 | .48 | 45 | .33 | 61 | .68 | 59 |
| How many fruit juices did you drink yesterday? *Regular cartons (330 ml)* | .18 | 96 | .33 | 92 | .32 | 68 | .26 | 70 | -.05 | 92 | .31 | 87 |
| I think that drinking fruit juices is… | .58 | 69 | .49 | 76 | .51 | 61 | .54 | 58 | .53 | 61 | .59 | 59 |
| I think it is recommended for children my age… | .70 | 78 | .45 | 65 | .40 | 62 | .60 | 70 | .33 | 63 | .38 | 60 |
| I think drinking fruit juices will make me fat. | .33 | 56 | .37 | 55 | .43 | 50 | .47 | 56 | .50 | 57 | .52 | 54 |
| I am allowed to take fruit juices whenever I want. | .59 | 59 | .62 | 56 | .64 | 58 | .73 | 69 | .78 | 61 | .65 | 56 |
| Do your parents/care givers have rules about how many fruit juices you are allowed to drink? | .71 | 90 | .52 | 77 | .60 | 88 | .58 | 90 | .76 | 91 | .67 | 85 |
| Are there usually fruit juices in your home? | .71 | 65 | .48 | 56 | .75 | 65 | .65 | 57 | .72 | 62 | .71 | 50 |
| In which situations are you most likely to drink fruit juices? *During the weekend* | .37 | 68 | .41 | 70 | .34 | 66 | .54 | 77 | .00 | 61 | .41 | 71 |
| In which situations are you most likely to drink fruit juices? *Breakfast* | .58 | 81 | .50 | 76 | .37 | 70 | .59 | 80 | .00 | 93 | .60 | 79 |
| In which situations are you most likely to drink fruit juices? *Lunch* | .54 | 88 | .56 | 81 | .43 | 77 | .29 | 66 | .00 | 48 | .32 | 78 |
| In which situations are you most likely to drink fruit juices? *Dinner* | .54 | 88 | .38 | 83 | .33 | 71 | .55 | 87 | .00 | 44 | .17 | 78 |
| In which situations are you most likely to drink fruit juices? *At school* | .61 | 90 | .49 | 85 | .53 | 77 | .60 | 81 | .00 | 50 | .49 | 76 |
| In which situations are you most likely to drink fruit juices? *While watching television* | .52 | 88 | .49 | 86 | .42 | 76 | .53 | 82 | .00 | 35 | .45 | 78 |
| In which situations are you most likely to drink fruit juices? *As a thirst quencher between meals* | .46 | 78 | .33 | 79 | .53 | 76 | .36 | 71 | .00 | 77 | .31 | 76 |
| In which situations are you most likely to drink fruit juices? *During/after sports* | .37 | 80 | .48 | 79 | .46 | 76 | .58 | 80 | .00 | 44 | .30 | 70 |
| In which situations are you most likely to drink fruit juices? *When I am with friends* | .39 | 78 | .45 | 74 | .27 | 66 | .50 | 82 | .00 | 60 | .31 | 83 |
| In which situations are you most likely to drink fruit juices? *At birthdays/parties* | .40 | 72 | .25 | 63 | .54 | 77 | .50 | 80 | .00 | 50 | .29 | 64 |
| In which situations are you most likely to drink fruit juices? *I never drink fruit juices* | .39 | 97 | -.01 | 98 | .39 | 98 | .32 | 97 | .00 | 86 | .00 | 99 |
| From Monday to Friday during school weeks, on how many days do you usually eat breakfast? | .84 | 86 | .68 | 71 | .81 | 77 | .61 | 90 | .32 | 84 | .67 | 92 |
| On how many days in the weekend days (Saturday and Sunday) do you usually eat breakfast? | .58 | 91 | .69 | 89 | .69 | 89 | .63 | 91 | .45 | 85 | .16 | 24 |
| What do you usually have for breakfast on school days? | .43 | 82 | .62 | 61 | .43 | 69 | .19 | 77 | .19 | 83 | .92 | 91 |
| What is the reason that you usually skip breakfast? | .81 | 86 | .66 | 77 | .75 | 78 | .74 | 86 | .80 | 86 | .66 | 86 |
| Did you eat breakfast yesterday? | .74 | 96 | .55 | 86 | .64 | 84 | .65 | 97 | .19 | 93 | .64 | 95 |
| Did you eat lunch yesterday? | .00 | 98 | .00 | 98 | .25 | 92 | .32 | 88 | .42 | 89 | - | 100 |
| Did you eat dinner yesterday? | .23 | 94 | .44 | 86 | .04 | 86 | -.02 | 97 | .00 | 100 | .66 | 98 |
| Did you eat anything between meals yesterday? | .45 | 79 | .42 | 72 | .28 | 72 | .43 | 83 | .41 | 70 | .28 | 79 |
| I think that eating breakfast is… | .68 | 87 | .43 | 77 | .71 | 65 | .47 | 77 | .38 | 82 | .41 | 74 |
| I think it is recommended for children of my age to… | .44 | 76 | .41 | 68 | .21 | 65 | .20 | 76 | .48 | 91 | .62 | 78 |
| I think not eating breakfast will make me fat. | .25 | 43 | .44 | 49 | .42 | 61 | .47 | 45 | .41 | 56 | .36 | 67 |
| I think eating breakfast will make me fat. | .39 | 53 | .51 | 54 | .44 | 56 | .42 | 51 | .41 | 61 | .58 | 55 |
| If I eat breakfast, my parents/care givers think this is… | .51 | 80 | .57 | 79 | .48 | 69 | .67 | 76 | .20 | 78 | .66 | 84 |
| If I eat breakfast, most of my friends think this is… | .56 | 76 | .63 | 63 | .46 | 56 | .57 | 62 | .40 | 62 | .46 | 59 |
| How often do your parents/care givers eat breakfast? | .72 | 75 | .63 | 52 | .72 | 66 | .71 | 83 | .60 | 81 | .65 | 83 |
| How often do most of your friends eat breakfast? | .47 | 65 | .54 | 54 | .43 | 60 | .44 | 64 | .48 | 67 | .51 | 73 |
| I like eating breakfast. | .69 | 82 | .45 | 60 | .73 | 62 | .65 | 72 | .77 | 85 | .61 | 78 |
| Eating breakfast is something that I do without even really thinking about it. | .65 | 62 | .57 | 48 | .51 | 46 | .57 | 53 | .66 | 60 | .53 | 58 |
| I find eating breakfast every day… | .78 | 69 | .57 | 57 | .69 | 48 | .74 | 70 | .76 | 81 | .67 | 71 |
| My parents/care givers encourage me to have breakfast. | .61 | 59 | .54 | 57 | .43 | 62 | .70 | 53 | .55 | 59 | .77 | 68 |
| Do your parents/care givers have rules about whether you should eat breakfast? | .51 | 78 | .48 | 75 | .56 | 83 | .61 | 80 | .66 | 83 | .47 | 73 |
| If you ask your parents/care givers to buy a certain brand of food or drink for breakfast, will they do it? | .46 | 53 | .47 | 56 | .36 | 51 | .59 | 61 | .58 | 66 | .45 | 47 |
| Are there usually breakfast products (milk, cereals, bread etc) at your home? | .43 | 77 | .46 | 74 | .43 | 72 | .52 | 80 | .30 | 64 | .21 | 80 |
| How often do you eat breakfast with your parents/care givers? | .81 | 66 | .63 | 38 | .66 | 5 | .84 | 69 | .54 | 53 | .73 | 56 |
| In which situations do you usually eat your breakfast? *At a set table at home* | .60 | 96 | .68 | 93 | .65 | 83 | .53 | 91 | .00 | 95 | .70 | 92 |
| In which situations do you usually eat your breakfast? *In bed* | - | 100 | .50 | 94 | .51 | 86 | .39 | 98 | - | - | .79 | 98 |
| In which situations do you usually eat your breakfast? *While watching television* | .61 | 92 | .57 | 88 | .43 | 77 | .74 | 88 | .00 | 62 | .67 | 85 |
| In which situations do you usually eat your breakfast? *On my way to school* | 1.00 | 100 | .24 | 93 | -.02 | 94 | .83 | 99 | .00 | 25 | .00 | 99 |
| In which situations do you usually eat your breakfast? *At school before the class starts* | .00 | 99 | -.02 | 96 | .52 | 85 | -.02 | 96 | - | - | -.01 | 98 |
| In which situations do you usually eat your breakfast? *I never eat breakfast* | .67 | 99 | .40 | 95 | .38 | 90 | .00 | 99 | - | - | 1.00 | 100 |
| How many days do you usually bike to school? | .92 | 85 | .51 | 93 | .92 | 87 | .98 | 91 | .88 | 44 | .90 | 92 |
| If you bike to school, how long does it take you to bike to school? | .81 | 85 | .47 | 83 | .75 | 85 | .89 | 89 | .81 | 78 | .56 | 87 |
| How many days a week do you usually walk to school? | .92 | 84 | .91 | 78 | .87 | 76 | .99 | 89 | .75 | 75 | .94 | 85 |
| If you walk to school, how long does it take you to walk to school? | .58 | 77 | .62 | 79 | .76 | 74 | .65 | 75 | .75 | 75 | .68 | 62 |
| How many days do you usually travel by car to school? | .94 | 86 | .91 | 79 | .83 | 78 | .80 | 85 | .97 | 89 | .90 | 88 |
| How many days do you usually travel by public transport (bus, school bus, tram, metro) to school? | .11 | 96 | .54 | 90 | .84 | 79 | .00 | 99 | .97 | 94 | .96 | 96 |
| How did you go to school today? | .69 | 78 | .73 | 91 | .82 | 83 | .57 | 86 | .62 | 70 | .88 | 88 |
| What do you usually do during breaks at school? | .71 | 85 | .73 | 87 | .58 | 76 | .75 | 89 | .82 | 90 | .92 | 91 |
| I do not participate in any sports activities | .70 | 92 | .64 | 87 | .55 | 92 | .93 | 99 | .00 | 85 | .90 | 95 |
| In a total week how many hours do you do this sport? | .81 | 56 | .54 | 51 | .70 | 49 | .78 | 62 | .83 | 55 | .75 | 52 |
| I do not have a second sport | .75 | 87 | .53 | 78 | .49 | 87 | .74 | 87 | .00 | 55 | .74 | 88 |
| In a total week how many hours do you do this sport? | 1.00 | 58 | 1.00 | 52 | 1.00 | 19 | 1.00 | 55 | 1.00 | 43 | 1.00 | 48 |
| How many hours of sports did you do yesterday? | .23 | 32 | .35 | 50 | .10 | 21 | .34 | 43 | .06 | 25 | -.12 | 29 |
| I think that physical activity/sports is...... | .54 | 91 | .18 | 92 | .58 | 71 | .50 | 84 | .62 | 87 | .25 | 90 |
| I think it is recommended for children of my age...... | .37 | 51 | .42 | 45 | .46 | 43 | .44 | 52 | .62 | 51 | .43 | 49 |
| I think not doing physical activity/sports will make me fat. | .41 | 48 | .44 | 52 | .58 | 63 | .37 | 60 | .46 | 48 | .55 | 55 |
| If I do physical activity/sports, my parents/care givers think this is...... | .37 | 81 | .22 | 88 | .52 | 77 | .61 | 83 | .22 | 75 | .46 | 81 |
| If I do physical activity/sports, most of my friends think this is...... | .58 | 70 | .55 | 77 | .64 | 67 | .72 | 67 | .38 | 66 | .40 | 71 |
| How often do your parents/care givers do physical activity/sports? | .58 | 60 | .71 | 57 | .51 | 57 | .72 | 58 | .71 | 63 | .64 | 52 |
| How often do most of your friends do physical activity/sports? | .34 | 72 | .42 | 62 | .64 | 59 | .44 | 66 | .68 | 75 | .54 | 69 |
| I like doing physical activity/sports. | .63 | 87 | .48 | 84 | .68 | 70 | .65 | 86 | .66 | 74 | .37 | 87 |
| Physical activity/sports is something that I do without even really thinking about it. | .67 | 59 | .50 | 53 | .58 | 53 | .50 | 52 | .56 | 49 | .55 | 43 |
| I find doing physical activity/sports for 1 hour every day...... | .66 | 73 | .56 | 74 | .81 | 78 | .55 | 65 | .64 | 65 | .40 | 67 |
| My parents/care givers encourage me to be physically active/do sports. | .62 | 57 | .61 | 67 | .58 | 74 | .71 | 62 | .57 | 48 | .67 | 63 |
| My parents/care givers help me if I need something for my sports. | .77 | 83 | .60 | 75 | .43 | 64 | .62 | 81 | .63 | 78 | .54 | 73 |
| Do your parents/care givers have rules about whether you should be physically active/do sports? | .61 | 83 | .44 | 72 | .26 | 74 | .51 | 80 | .43 | 78 | .42 | 74 |
| Do your parents/care givers allow you to take part in physical activity/do sports? | .38 | 95 | .00 | 98 | .25 | 86 | .32 | 97 | -.03 | 94 | -.04 | 92 |
| If you indicate that you like a certain physical activity/sports will your parents/care givers allow you to do it? | .68 | 56 | .52 | 60 | .66 | 72 | .57 | 60 | .72 | 69 | .48 | 61 |
| Do you have the following things at home that you can use for physical activities/sports? *Bike* | .65 | 96 | .51 | 87 | .51 | 84 | .44 | 92 | .00 | 96 | .75 | 97 |
| Do you have the following things at home that you can use for physical activities/sports? *Tennis and or badminton racket* | .71 | 91 | .69 | 85 | .63 | 82 | .49 | 83 | .00 | 81 | .76 | 91 |
| Do you have the following things at home that you can use for physical activities/sports? *Ball* | .69 | 94 | .54 | 90 | .47 | 84 | .31 | 94 | .00 | 88 | .48 | 95 |
| Do you have the following things at home that you can use for physical activities/sports? *Sporting shoes* | .46 | 89 | .49 | 90 | .55 | 83 | .58 | 91 | .00 | 87 | .38 | 91 |
| Do you have the following things at home that you can use for physical activities/sports? *Skipping rope* | .77 | 93 | .77 | 88 | .70 | 85 | .77 | 88 | .00 | 81 | .87 | 95 |
| Do you have the following things at home that you can use for physical activities/sports? *Skates* | .86 | 96 | .70 | 88 | .73 | 87 | .70 | 91 | .00 | 81 | .80 | 94 |
| Do you have the following things at home that you can use for physical activities/sports? *Skies* | .74 | 96 | .51 | 94 | .72 | 94 | .73 | 93 | .00 | 89 | .79 | 92 |
| Do you have the following things at home that you can use for physical activities/sports? *Skate board* | .81 | 91 | .81 | 92 | .75 | 88 | .65 | 83 | .00 | 82 | .75 | 89 |
| How often do you take part in physical activity/do sports with your parents/care givers? | .45 | 53 | .29 | 43 | .54 | 49 | .60 | 33 | .48 | 56 | .43 | 38 |
| About how many hours a day do you usually watch television in your free time? Week days (average of all weekdays) | .70 | 45 | .65 | 41 | .67 | 42 | .67 | 47 | .61 | 45 | .56 | 33 |
| About how many hours a day do you usually watch television in your free time? Weekend days (average of all weekend days) | .70 | 36 | .66 | 41 | .62 | 33 | .66 | 41 | .70 | 41 | .70 | 26 |
| About how many hours a day do you usually play games on a computer, or use your computer for leisure activities in your free time? Week days (average of all week days) | .48 | 31 | .65 | 45 | .75 | 41 | .60 | 41 | .67 | 45 | .72 | 48 |
| About how many hours a day do you usually play games on a computer, or use your computer for leisure activities in your free time? Weekend days (average of all weekend days) | .53 | 29 | .68 | 37 | .76 | 42 | .67 | 37 | .58 | 36 | .63 | 35 |
| About how many hours did you watch television yesterday? | .70 | 44 | .60 | 29 | .71 | 34 | .67 | 34 | .64 | 37 | .61 | 43 |
| About how many hours did you play games on a computer, games console or use your computer for leisure activities yesterday? | .53 | 48 | .43 | 41 | .64 | 32 | .50 | 35 | .53 | 33 | .31 | 38 |
| I think watching television is… | .59 | 66 | .48 | 66 | .71 | 58 | .58 | 71 | .65 | 62 | .38 | 79 |
| I think it is recommended for children of my age… | .48 | 49 | .44 | 44 | .46 | 39 | .42 | 52 | .57 | 51 | .55 | 43 |
| I think watching too much television can help making me fat. | .64 | 52 | .66 | 57 | .60 | 57 | .66 | 58 | .63 | 53 | .62 | 57 |
| If I watch television, my parents/care givers think this is… | .53 | 73 | .28 | 61 | .63 | 65 | .37 | 71 | .71 | 77 | .55 | 70 |
| If I watch television, most of my friends think this is… | .56 | 70 | .47 | 57 | .63 | 66 | .49 | 70 | .68 | 79 | .59 | 60 |
| How often do your parents/care givers watch television? | .68 | 67 | .64 | 57 | .75 | 72 | .65 | 67 | .69 | 67 | .56 | 46 |
| How often do most of your friends watch television? | .49 | 65 | .40 | 63 | .51 | 65 | .43 | 61 | .42 | 69 | .39 | 48 |
| I like watching television | .60 | 64 | .57 | 51 | .71 | 69 | .66 | 65 | .71 | 67 | .70 | 66 |
| Watching television is something that I do without even really thinking about | .70 | 53 | .63 | 57 | .53 | 13 | .64 | 57 | .71 | 53 | .65 | 59 |
| I find not watching television… | .58 | 56 | .58 | 50 | .73 | 58 | .57 | 45 | .70 | 57 | .63 | 53 |
| My parents/care givers allow me to watch television whenever I want. | .72 | 47 | .64 | 50 | .48 | 48 | .70 | 53 | .79 | 62 | .52 | 43 |
| If I ask my parents/care givers to watch television, I can do so. | .64 | 60 | .49 | 53 | .53 | 62 | .62 | 62 | .73 | 68 | .51 | 60 |
| Do your parents/care givers have rules about how many hours per day you are allowed to watch television? | .61 | 81 | .50 | 79 | .46 | 75 | .57 | 79 | .74 | 87 | .64 | 82 |
| Do you have a television in your own bedroom? | .97 | 99 | .92 | 96 | .74 | 92 | .96 | 98 | .91 | 96 | .86 | 94 |
| How often do you watch television with your parents/care givers? | .68 | 48 | .55 | 41 | .62 | 44 | .65 | 35 | .62 | 51 | .63 | 37 |
| How often do you watch television during meals? *Breakfast* | .81 | 78 | .44 | 70 | .69 | 57 | .85 | 72 | .83 | 66 | .78 | 68 |
| How often do you watch television during meals? *Lunch* | .76 | 72 | .67 | 52 | .70 | 58 | .77 | 61 | .72 | 58 | .82 | 64 |
| How often do you watch television during meals? *Dinner* | .79 | 65 | .55 | 43 | .79 | 55 | .73 | 64 | .82 | 65 | .75 | 57 |
| Do you think you are too thin or too fat? | .88 | 84 | .84 | 77 | .86 | 80 | .88 | 85 | .89 | 93 | .88 | 85 |
| How often have you tried to get slimmer/thinner during the last year? | .80 | 80 | .77 | 75 | .84 | 75 | .90 | 84 | .57 | 19 | .63 | 74 |
| Do you try to get slimmer or thinner right now? | .67 | 87 | .73 | 86 | .73 | 92 | .81 | 92 | .53 | 90 | .64 | 87 |
