# Supplementary material for: Test-retest reliability and construct validity of the ENERGY-child questionnaire on energy balance-related behaviours and their potential determinants: the ENERGY-project
Source: Int J Behav Nutr Phys Act. 2011 Dec 9;8:136. doi: 10.1186/1479-5868-8-136 (PMC3293727; doi:10.1186/1479-5868-8-136)
Supplement: Additional file 2 — Table. Country-specific results of the construct validity study of the ENERGY-child questionnaire: agreement (per questionnaire item) between questionnaire and interview as indicated by intraclass correlation coefficients (ICC) and percentage agreement (agree). [file 1479-5868-8-136-S2.DOC]

**Additional file 2.**

**Table.** Country-specific results of the construct validity study of the ENERGY-child questionnaire: agreement (per questionnaire item) between questionnaire and interview as indicated by intraclass correlation coefficients (ICC) and percentage agreement (agree).

| item/country | Belgium | | Greece | | Hungary | | Netherlands | | Norway | | Spain | |
| --- | --- | --- | --- | --- | --- | --- | --- | --- | --- | --- | --- | --- |
|  | ICC | agree | ICC | agree | ICC | agree | ICC | agree | ICC | agree | ICC | agree |
| How many times a week do you usually drink fizzy drinks and fruit squash? | .80 | 40 | .99 | 94 | .49 | 44 | -.14 | 45 | .18 | 36 | .62 | 67 |
| On a day that you drink fizzy drinks and fruit squash, how many glasses, cans or bottles do you drink on such a day? *Glasses or small bottles (250 ml)* | .40 | 60 | .84 | 88 | - | - | -.08 | 40 | .41 | 67 | -.17 | 40 |
| On a day that you drink fizzy drinks and fruit squash, how many glasses, cans or bottles do you drink on such a day? *Cans (330 ml)* | .61 | 87 | .79 | 81 | - | - | .11 | 65 | .55 | 77 | .28 | 47 |
| On a day that you drink fizzy drinks and fruit squash, how many glasses, cans or bottles do you drink on such a day? *Bottles (500 ml)* | -.03 | 87 | - | 100 | - | - | .00 | 75 | .00 | 67 | .00 | 73 |
| How many fizzy drinks or fruit squash did you drink yesterday? *Glasses or small bottles (250 ml)* | .38 | 67 | .94 | 100 | - | - | -.10 | 30 | .44 | 67 | .79 | 73 |
| How many fizzy drinks or fruit squash did you drink yesterday? *Cans (330 ml)* | .00 | 87 | 1.00 | 100 | - | - | .04 | 65 | .00 | 92 | .65 | 93 |
| How many fizzy drinks or fruit squash did you drink yesterday? *Bottles (500 ml)* | .00 | 93 | 1.00 | 100 | - | - | .47 | 90 | -.10 | 83 | .00 | 93 |
| I think that drinking fizzy drinks or fruit squash is...... | .44 | 27 | .26 | 75 | .11 | 18 | .50 | 40 | -.26 | - | .15 | 60 |
| I think drinking fizzy drinks or fruit squash will make me fat | .52 | 71 | .84 | 69 | 13 | 40 | .26 | 45 | .53 | 13 | .45 | 33 |
| If I drink fizzy drinks or fruit squash, my parents/care givers think this is...... | .55 | 57 | .85 | 81 | 49 | 25 | -.16 | 45 | .40 | 40 | .21 | 53 |
| If I drink fizzy drinks or fruit squash, most of my friends think this is...... | -.06 | 58 | .64 | 60 | .22 | 50 | .29 | 45 | -.33 | 40 | .28 | 40 |
| How often do your parents/care givers drink fizzy drinks or fruit squash? | 1.00 | 47 | 1.00 | 69 | 1.00 | 50 | 1.00 | 42 | 1.00 | 46 | 1.00 | 53 |
| How often do most of your friends drink fizzy drinks or fruit squash? | .29 | 57 | .33 | 64 | .60 | 70 | -.11 | 60 | .00 | 73 | .55 | 43 |
| I like the taste of fizzy drinks or fruit squash. | .37 | 60 | .68 | 69 | -.13 | 20 | .10 | 85 | .37 | 60 | .41 | 47 |
| Drinking fizzy drinks or fruit squash is something that I do without even really thinking about it. | -.13 | 27 | .62 | 19 | .62 | 50 | .09 | 40 | -.68 | 27 | .33 | 40 |
| I find drinking no fizzy drinks or fruit squash...... | .50 | 36 | .70 | 56 | .04 | 25 | -.10 | 25 | .21 | 27 | -.13 | 21 |
| If I ask my parents/care givers for a fizzy drink or fruit squash, I get one. | .56 | 40 | .61 | 56 | .00 | 50 | .58 | 50 | .00 | 43 | .44 | 27 |
| I am allowed to take fizzy drinks or fruit squash whenever I want. | .29 | 20 | .78 | 81 | -1.00 | 0 | .33 | 45 | -.09 | 28 | .20 | 43 |
| Do your parents/care givers have rules about how many fizzy drinks or fruit squash you are allowed to drink? | .30 | 67 | 1.00 | 100 | .67 | 82 | .46 | 70 | .30 | 64 | .08 | 67 |
| If you ask your parents/care givers to buy a certain brand of fizzy drinks or fruit squash, will she do it? | .15 | 40 | .91 | 67 | .66 | 33 | .41 | 40 | .06 | 0 | .45 | 14 |
| Are there usually fizzy drinks or fruit squash at your home? | .47 | 50 | .85 | 56 | .11 | 27 | .27 | 60 | .58 | 36 | .11 | 33 |
| In which situations do you usually drink fizzy drinks or fruit squash? *During the weekend* | .74 | 87 | .88 | 94 | -.22 | 50 | .00 | 35 | -.22 | 50 | .26 | 60 |
| In which situations do you usually drink fizzy drinks or fruit squash? *Breakfast* | .00 | 93 | - | 100 | - | 100 | - | 100 | - | 100 | .00 | 93 |
| In which situations do you usually drink fizzy drinks or fruit squash? *Lunch* | .61 | 87 | - | 100 | .00 | 100 | .00 | 80 | - | 100 | .46 | 87 |
| In which situations do you usually drink fizzy drinks or fruit squash? *Dinner* | .00 | 87 | 1.00 | 100 | .00 | 100 | .27 | 65 | .00 | 80 | .00 | 80 |
| In which situations do you usually drink fizzy drinks or fruit squash? *At school* | .00 | 100 | - | 100 | .10 | 60 | -.08 | 85 | - | 100 | - | 100 |
| In which situations do you usually drink fizzy drinks or fruit squash? *While watching television* | .65 | 93 | 1.00 | 100 | .00 | 100 | -.02 | 45 | .00 | 90 | .46 | 87 |
| In which situations do you usually drink fizzy drinks or fruit squash? *As a thirst quencher between meals* | -.11 | 80 | 1.00 | 100 | .00 | 100 | .28 | 80 | .00 | 80 | .65 | 93 |
| In which situations do you usually drink fizzy drinks or fruit squash? *During/after sports* | .26 | 73 | - | 100 | .57 | 80 | .15 | 65 | - | 100 | .42 | 73 |
| In which situations do you usually drink fizzy drinks or fruit squash? *When I am with friends* | .47 | 73 | .88 | 94 | -.47 | 30 | .00 | 50 | .00 | 70 | .22 | 67 |
| In which situations do you usually drink fizzy drinks or fruit squash? *At birthdays/parties* | .49 | 80 | .68 | 88 | .10 | 60 | .07 | 70 | .40 | 80 | .11 | 53 |
| In which situations do you usually drink fizzy drinks or fruit squash? *I never drink fizzy drinks or fruit squash* | .65 | 93 | - | 100 | - | 100 | .00 | 90 | - | 100 | .00 | 93 |
| How often do you spend your own money on fizzy drinks or fruit squash? | .61 | 80 | .88 | 75 | .19 | 36 | .10 | 45 | -.06 | 20 | .38 | 60 |
| If the price of fizzy drinks and fruit squash were doubled, I would buy less fizzy drinks or fruit squash from my own money. | .24 | 57 | .85 | 50 | .31 | 29 | .19 | 35 | .57 | 47 | .53 | 33 |
| How many times a week do you usually drink fruit juices? | .33 | 36 | .94 | 81 | - | 100 | .65 | 42 | .77 | 43 | .86 | 60 |
| On a day that you drink fruit juices, how many glasses or cartons do you drink on such a day? *Glasses or small cartons (250 ml)* | .48 | 53 | .47 | 81 | - | - | .51 | 25 | .32 | 36 | .72 | 67 |
| On a day that you drink fruit juices, how many glasses or cartons do you drink on such a day? *Regular cartons (330 ml)* | - | 100 | 1.00 | 100 | - | - | .04 | 45 | -.13 | 71 | -.08 | 80 |
| How many fruit juices did you drink yesterday? *Glasses or small cartons (250 ml)* | .57 | 60 | .92 | 88 | - | 100 | .66 | 50 | .18 | 54 | .66 | 60 |
| How many fruit juices did you drink yesterday? *Regular cartons (330 ml)* | - | 100 | .62 | 88 | .00 | 33 | .04 | 70 | .00 | 93 | -.11 | 80 |
| I think that drinking fruit juices is… | -.27 | 40 | .44 | 75 | -.11 | 33 | .03 | 60 | -.01 | 40 | .20 | 47 |
| I think it is recommended for children my age… | -.14 | 50 | .55 | 71 | .39 | 67 | .31 | 35 | .68 | 40 | .42 | 53 |
| I think drinking fruit juices will make me fat. | .18 | 33 | .84 | 63 | .40 | 46 | .03 | 30 | .35 | 60 | .53 | 53 |
| I am allowed to take fruit juices whenever I want. | .43 | 53 | .69 | 67 | .00 | 100 | .51 | 55 | .01 | 50 | .75 | 40 |
| Do your parents/care givers have rules about how many fruit juices you are allowed to drink? | .71 | 87 | .88 | 94 | .77 | 92 | .46 | 90 | .44 | 87 | .49 | 73 |
| Are there usually fruit juices in your home? | .87 | 60 | .75 | 87 | .38 | 27 | .37 | 35 | .56 | 39 | .37 | 67 |
| In which situations are you most likely to drink fruit juices? *During the weekend* | .30 | 67 | 1.00 | 100 | -.33 | 39 | .30 | 70 | .26 | 71 | .35 | 73 |
| In which situations are you most likely to drink fruit juices? *Breakfast* | .85 | 93 | 1.00 | 100 | .00 | 100 | .32 | 60 | .86 | 93 | .49 | 73 |
| In which situations are you most likely to drink fruit juices? *Lunch* | .46 | 87 | 1.00 | 100 | .25 | 70 | -.18 | 45 | .00 | 71 | .20 | 73 |
| In which situations are you most likely to drink fruit juices? *Dinner* | .30 | 80 | .85 | 94 | .00 | 100 | .07 | 70 | -.13 | 71 | .46 | 87 |
| In which situations are you most likely to drink fruit juices? *At school* | .00 | 87 | 1.00 | 100 | -.09 | 85 | .15 | 65 | - | 100 | .65 | 93 |
| In which situations are you most likely to drink fruit juices? *While watching television* | .00 | 93 | 1.00 | 100 | .33 | 77 | .19 | 70 | .00 | 79 | .00 | 87 |
| In which situations are you most likely to drink fruit juices? *As a thirst quencher between meals* | -.11 | 80 | - | 100 | -.14 | 46 | .23 | 75 | .32 | 71 | -.47 | 40 |
| In which situations are you most likely to drink fruit juices? *During/after sports* | .00 | 87 | 1.00 | 100 | .00 | 100 | .12 | 70 | .00 | 86 | -.27 | 47 |
| In which situations are you most likely to drink fruit juices? *When I am with friends* | .83 | 93 | 1.00 | 100 | .60 | 85 | -.09 | 80 | .00 | 93 | .00 | 80 |
| In which situations are you most likely to drink fruit juices? *At birthdays/parties* | .08 | 67 | 1.00 | 100 | .05 | 54 | .00 | 70 | .00 | 93 | .42 | 87 |
| In which situations are you most likely to drink fruit juices? *I never drink fruit juices* | 1.00 | 100 | - | 100 | - | 100 | .00 | 95 | 1.00 | 100 | - | 100 |
| From Monday to Friday during school weeks, on how many days do you usually eat breakfast? | .81 | 93 | .76 | 88 | .60 | 50 | .00 | 70 | -.03 | 64 | .88 | 100 |
| On how many days in the weekend days (Saturday and Sunday) do you usually eat breakfast? | .65 | 87 | 1.00 | 100 | .66 | 86 | .46 | 90 | .00 | 73 | -.11 | 79 |
| What do you usually have for breakfast on school days? | .00 | 67 | .83 | 88 | .84 | 83 | .18 | 42 | -.17 | 73 | .70 | 80 |
| What is the reason that you usually skip breakfast? | .25 | 80 | 1.00 | 100 | .29 | 33 | .16 | 45 | .52 | 85 | .76 | 87 |
| Did you eat breakfast yesterday? | .00 | 93 | - | 100 | .18 | 73 | -.08 | 85 | 1.00 | 100 | .00 | 93 |
| Did you eat lunch yesterday? | - | 100 | - | 100 | - | 100 | .00 | 95 | .00 | 77 | - | 100 |
| Did you eat dinner yesterday? | 1.00 | 100 | - | 100 | - | 100 | - | 100 | - | 100 | 1.00 | 100 |
| Did you eat anything between meals yesterday? | .35 | 67 | 1.00 | 100 | - | - | .36 | 85 | -.07 | 46 | .00 | 85 |
| I think that eating breakfast is… | .26 | 47 | .21 | 63 | -.18 | 46 | .07 | 25 | .39 | 57 | -.32 | 33 |
| I think it is recommended for children of my age to… | .25 | 77 | .92 | 93 | .00 | 75 | -.01 | 65 | .00 | 87 | .30 | 67 |
| I think not eating breakfast will make me fat. | .10 | 40 | .76 | 38 | - | - | 1.00 | 100 | -.11 | 15 | .17 | 36 |
| I think eating breakfast will make me fat. | .38 | 33 | .70 | 44 | .31 | 13 | .13 | 30 | .01 | 47 | .25 | 7 |
| If I eat breakfast, my parents/care givers think this is… | .12 | 43 | -.25 | 63 | -.26 | 53 | .29 | 35 | .59 | 67 | -.06 | 53 |
| If I eat breakfast, most of my friends think this is… | .55 | 53 | .63 | 80 | .17 | 39 | -.17 | 45 | .14 | 27 | .64 | 80 |
| How often do your parents/care givers eat breakfast? | .72 | 80 | .88 | 69 | .46 | 57 | .05 | 80 | .71 | 58 | .34 | 87 |
| How often do most of your friends eat breakfast? | .44 | 53 | .42 | 17 | .49 | 55 | .08 | 50 | -.15 | 27 | .40 | 53 |
| I like eating breakfast. | -.24 | 60 | -.24 | 43 | .00 | 60 | .37 | 55 | .00 | 50 | .59 | 73 |
| Eating breakfast is something that I do without even really thinking about it. | .30 | 40 | .76 | 56 | -.15 | 57 | -.11 | 40 | .00 | 21 | .10 | 36 |
| I find eating breakfast every day… | .44 | 36 | .58 | 56 | .44 | 27 | .07 | 30 | .25 | 73 | .50 | 47 |
| My parents/care givers encourage me to have breakfast. | .34 | 27 | .62 | 75 | .00 | 71 | .34 | 55 | .11 | 33 | .46 | 64 |
| Do your parents/care givers have rules about whether you should eat breakfast? | .11 | 53 | 1.00 | 100 | .36 | 64 | .11 | 55 | .62 | 80 | .06 | 53 |
| If you ask your parents/care givers to buy a certain brand of food or drink for breakfast, will they do it? | -.10 | 47 | .45 | 44 | .24 | 50 | -.39 | 45 | .41 | 64 | .54 | 60 |
| Are there usually breakfast products (milk, cereals, bread etc) at your home? | .08 | 60 | .79 | 88 | - | 100 | -.15 | 75 | .00 | 80 | .37 | 80 |
| How often do you eat breakfast with your parents/care givers? | .83 | 69 | .94 | 81 | .16 | 13 | .34 | 15 | .76 | 43 | .77 | 47 |
| In which situations do you usually eat your breakfast? *At a set table at home* | - | 100 | - | 100 | .34 | 80 | -.19 | 70 | .29 | 79 | .00 | 93 |
| In which situations do you usually eat your breakfast? *In bed* | - | 100 | - | 100 | .65 | 93 | - | 100 | 1.00 | 100 | .00 | 93 |
| In which situations do you usually eat your breakfast? *While watching television* | - | 100 | - | 100 | .61 | 87 | -.13 | 55 | .45 | 79 | .65 | 93 |
| In which situations do you usually eat your breakfast? *On my way to school* | .00 | 93 | - | 100 | - | 100 | .00 | 95 | - | 100 | - | 100 |
| In which situations do you usually eat your breakfast? *At school before the class starts* | - | 100 | - | 100 | -.12 | 73 | - | 100 | - | 100 | - | 100 |
| In which situations do you usually eat your breakfast? *I never eat breakfast* | - | 100 | - | 100 | - | 100 | .00 | 95 | - | 100 | - | 100 |
| How many days do you usually bike to school? | .96 | 79 | - | 100 | -.04 | 60 | .41 | 40 | .82 | 57 | 1.00 | 100 |
| If you bike to school, how long does it take you to bike to school? | .58 | 77 | - | 100 | .22 | 80 | .46 | 50 | .49 | 53 | 1.00 | 100 |
| How many days a week do you usually walk to school? | .90 | 79 | 1.00 | 100 | -.08 | 67 | .69 | 50 | .87 | 64 | .83 | 93 |
| If you walk to school, how long does it take you to walk to school? | .82 | 85 | .97 | 94 | .57 | 83 | -.01 | 45 | .57 | 67 | .89 | 80 |
| How many days do you usually travel by car to school? | .65 | 64 | 1.00 | 100 | - | 100 | .39 | 70 | .95 | 93 | .77 | 73 |
| How many days do you usually travel by public transport (bus, school bus, tram, metro) to school? | - | 100 | - | 100 | - | 100 | .00 | 95 | .00 | 92 | .77 | 93 |
| How did you go to school today? | .79 | 92 | 1.00 | 100 | - | - | .45 | 45 | .12 | 64 | .80 | 80 |
| What do you usually do during breaks at school? | .45 | 57 | .96 | 94 | .53 | 80 | -.14 | 75 | .83 | 93 | .68 | 86 |
| I do not participate in any sports activities | .65 | 93 | 1.00 | 100 | - | 100 | 1.00 | 100 | .50 | 67 | .73 | 86 |
| In a total week how many hours do you do this sport? | .85 | 36 | 1.00 | 100 | .66 | 43 | .07 | 17 | .12 | 50 | .57 | 83 |
| I do not have a second sport | .46 | 73 | 1.00 | 100 | - | 100 | .41 | 70 | .00 | 30 | .42 | 90 |
| In a total week how many hours do you do this sport? | 1.00 | 29 | 1.00 | 100 | 1.00 | 100 | 1.00 | - | 1.00 | - | 1.00 | 100 |
| How many hours of sports did you do yesterday? | .33 | 33 | 1.00 | 100 | .93 | 33 | -.04 | 60 | .05 | 33 | - | 100 |
| I think that physical activity/sports is...... | .00 | 60 | -.12 | 73 | -.08 | 79 | .21 | 60 | .00 | 93 | .10 | 47 |
| I think it is recommended for children of my age...... | .15 | 50 | .52 | 71 | .33 | 60 | -.41 | 20 | .04 | - | .11 | 50 |
| I think NOT doing physical activity/sports will make me fat. | .61 | 46 | .73 | 71 | .90 | 73 | -.12 | 35 | .64 | 50 | .36 | 73 |
| If I do physical activity/sports, my parents/care givers think this is...... | .13 | 53 | -.11 | 75 | .00 | 85 | -.21 | 15 | -.32 | 14 | .26 | 73 |
| If I do physical activity/sports, most of my friends think this is...... | .13 | 40 | .68 | 87 | .43 | 64 | .08 | 45 | .52 | 67 | .16 | 53 |
| How often do your parents/care givers do physical activity/sports? | .46 | 40 | .92 | 69 | .39 | 29 | -.18 | 40 | .84 | 62 | .71 | 47 |
| How often do most of your friends do physical activity/sports? | .47 | 60 | .62 | 50 | -.05 | 40 | .21 | 65 | .33 | 62 | -.10 | 47 |
| I like doing physical activity/sports. | -.08 | 86 | -.10 | 81 | .00 | 75 | .47 | 90 | -.11 | 79 | .24 | 67 |
| Physical activity/sports is something that I do without even really thinking about it. | -.37 | 13 | .19 | 53 | - | - | .13 | 50 | -.10 | 9 | .25 | 20 |
| I find doing physical activity/sports for 1 hour every day...... | -.04 | 20 | -.06 | 67 | -.35 | 33 | -.04 | 30 | .40 | 29 | .61 | 47 |
| My parents/care givers encourage me to be physically active/do sports. | .55 | 60 | .89 | 79 | - | - | .43 | 40 | .27 | 23 | .14 | 60 |
| My parents/care givers help me if I need something for my sports. | .13 | 50 | 1.00 | 100 | .00 | 87 | .07 | 80 | .00 | 77 | .73 | 80 |
| Do your parents/care givers have rules about whether you should be physically active/do sports? | .87 | 93 | 1.00 | 100 | .29 | 64 | .08 | 70 | .42 | 83 | .04 | 53 |
| Do your parents/care givers allow you to take part in physical activity/do sports? | - | 100 | 1.00 | 100 | - | 100 | - | 100 | - | 100 | - | 100 |
| If you indicate that you like a certain physical activity/sports will your parents/care givers allow you to do it? | .52 | 57 | .26 | 47 | -.26 | 50 | -.04 | 15 | .54 | 57 | .30 | 87 |
| Do you have the following things at home that you can use for physical activities/sports? *Bike* | -.11 | 79 | 1.00 | 100 | .65 | 92 | .66 | 95 | .00 | 86 | .00 | 93 |
| Do you have the following things at home that you can use for physical activities/sports? *Tennis and or badminton racket* | -.14 | 64 | 1.00 | 100 | .47 | 77 | -.23 | 30 | -.03 | 57 | .60 | 80 |
| Do you have the following things at home that you can use for physical activities/sports? *Ball* | .48 | 79 | 1.00 | 100 | .00 | 77 | -.09 | 75 | .00 | 64 | .00 | 87 |
| Do you have the following things at home that you can use for physical activities/sports? *Sporting shoes* | .38 | 71 | .65 | 94 | .07 | 31 | .00 | 20 | .00 | 64 | -.26 | 53 |
| Do you have the following things at home that you can use for physical activities/sports? *Skipping rope* | -.04 | 43 | 1.00 | 100 | .57 | 77 | .03 | 50 | .61 | 79 | .54 | 80 |
| Do you have the following things at home that you can use for physical activities/sports? *Skates* | .20 | 64 | 1.00 | 100 | .03 | 62 | .02 | 45 | .19 | 62 | .46 | 87 |
| Do you have the following things at home that you can use for physical activities/sports? *Skies* | .34 | 79 | 1.00 | 100 | .65 | 92 | -.09 | 75 | -.15 | 57 | .65 | 93 |
| Do you have the following things at home that you can use for physical activities/sports? *Skate board* | .38 | 64 | 1.00 | 100 | .28 | 77 | .00 | 50 | .71 | 85 | .46 | 73 |
| How often do you take part in physical activity/do sports with your parents/care givers? | -.14 | 47 | .85 | 69 | .19 | 50 | -.06 | 45 | .60 | 36 | .43 | 60 |
| About how many hours a day do you usually watch television in your free time? Week days (average of all weekdays) | .64 | 33 | .98 | 82 | .84 | 50 | .32 | 35 | .14 | 23 | .41 | 47 |
| About how many hours a day do you usually watch television in your free time? Weekend days (average of all weekend days) | .50 | 33 | .93 | 69 | .87 | 39 | .46 | 15 | .13 | 40 | .33 | 50 |
| About how many hours a day do you usually play games on a computer, or use your computer for leisure activities in your free time? Week days (average of all week days) | .23 | 27 | .96 | 94 | .14 | 14 | .09 | 5 | .46 | 27 | .30 | 33 |
| About how many hours a day do you usually play games on a computer, or use your computer for leisure activities in your free time? Weekend days (average of all weekend days) | .28 | 7 | 1.00 | 93 | .74 | 33 | .38 | 30 | .38 | 30 | .42 | 29 |
| About how many hours did you watch television yesterday? | .72 | 53 | .97 | 75 | .95 | 54 | -.04 | 20 | .37 | 33 | .47 | 33 |
| About how many hours did you play games on a computer, games console or use your computer for leisure activities yesterday? | .87 | 80 | .80 | 63 | - | - | .17 | 40 | .37 | 22 | -.29 | 33 |
| I think watching television is… | .19 | 33 | 1.00 | 100 | .33 | 15 | -.09 | 35 | -.02 | 27 | .39 | 47 |
| I think it is recommended for children of my age… | .16 | 7 | .91 | 67 | .37 | 36 | .02 | 25 | .25 | 13 | .48 | 40 |
| I think watching too much television can help making me fat. | .50 | 47 | .86 | 50 | .60 | 57 | .19 | 25 | .46 | 33 | .73 | 53 |
| If I watch television, my parents/care givers think this is… | .22 | 40 | .45 | 60 | .31 | 70 | -.30 | 55 | -.34 | 43 | .27 | 64 |
| If I watch television, most of my friends think this is… | .00 | 40 | .88 | 73 | -.11 | 33 | .05 | 35 | .17 | 50 | .67 | 60 |
| How often do your parents/care givers watch television? | .17 | 40 | .57 | 63 | .04 | 36 | .26 | 40 | .33 | 50 | .52 | 53 |
| How often do most of your friends watch television? | -.13 | 33 | .36 | 64 | .07 | 50 | .32 | 60 | .52 | 62 | .38 | 53 |
| I like watching television | .36 | 46 | .20 | 56 | .56 | 54 | .24 | 58 | .40 | 47 | .54 | 40 |
| Watching television is something that I do without even really thinking about | .32 | 47 | .72 | 60 | .06 | 40 | -.07 | 21 | .32 | 7 | .20 | 36 |
| I find not watching television… | .44 | 33 | .70 | 56 | .46 | 39 | .10 | 21 | .44 | 20 | .27 | 20 |
| My parents/care givers allow me to watch television whenever I want. | .25 | 20 | .85 | 56 | .34 | 31 | .30 | 32 | .23 | 20 | .04 | 40 |
| If I ask my parents/care givers to watch television, I can do so. | .21 | 47 | .52 | 75 | .34 | 60 | -.08 | 58 | .10 | 36 | .55 | 67 |
| Do your parents/care givers have rules about how many hours per day you are allowed to watch television? | .75 | 87 | .88 | 94 | .22 | 60 | .34 | 69 | .32 | 96 | .07 | 40 |
| Do you have a television in your own bedroom? | 1.00 | 100 | 1.00 | 100 | 1.00 | 100 | .41 | 70 | .86 | 93 | .85 | 93 |
| How often do you watch television with your parents/care givers? | .61 | 36 | .86 | 63 | .37 | 33 | .16 | 25 | .02 | 39 | .59 | 27 |
| How often do you watch television during meals? *Breakfast* | .84 | 85 | .95 | 88 | .92 | 73 | .49 | 35 | .27 | 46 | .74 | 73 |
| How often do you watch television during meals? *Lunch* | .64 | 69 | .91 | 63 | .63 | 80 | .11 | 35 | .77 | 64 | .61 | 47 |
| How often do you watch television during meals? *Dinner* | .69 | 42 | .69 | 63 | .71 | 54 | -.15 | 35 | .37 | 36 | .57 | 47 |
| Do you think you are too thin or too fat? | .62 | 86 | .84 | 69 | .65 | 69 | .18 | 40 | .87 | 93 | .56 | 87 |
| How often have you tried to get slimmer/thinner during the last year? | .50 | 54 | 1.00 | 100 | .80 | 90 | .40 | 55 | -.14 | 71 | .92 | 87 |
| Do you try to get slimmer or thinner right now? | .55 | 79 | 1.00 | 100 | 1.00 | 100 | .14 | 63 | .00 | 82 | .87 | 93 |
